# Materials of care: Engineering the future of the ICU

**DOI:** 10.2478/jccm-2025-0049

**Published:** 2025-10-31

**Authors:** Diana Portan

**Affiliations:** Department of Chemical Engineering, University of Patras, Patras, Greece; Department of Mechanical Engineering, Liaoning University of Technology, Jinzhou, China; Scientific Research and Technological Development Unit, George Emil Palade University of Medicine, Pharmacy, Science, and Technology of Targu Mures, Targu Mures, Romania

## Pairing materials, technologies and organs

In critical care, the line separating biology from technology is diminishing with progress. Organs that were once deemed beyond repair can now be supported, substituted, or even encouraged to heal. Artificial support is enabled through advanced materials and emerging technologies designed to imitate or enhance living tissue and function. This combination of organs and materials goes beyond mere mechanics; it signifies a profound merging of physiological principles and material design. As we improve these interfaces, the challenge lies in ensuring that innovation not only fulfils the need for survival but also upholds the integrity of the human body it aims to protect.

Initially, advanced materials were designed for: durability, resistance, and control [1]. Polymers, alloys, and composites were perfected to withstand impact rather than to support life. Later, they have been redirected toward healing, and for the ‘dialogue’ with living systems. Due to this shift, the use of technologies and advanced materials in critical care is revolutionizing patient care, enhancing diagnosis, treatment and safety [2]. Redefining the original goals of materials research, from protection to healing, has opened new paths for progress driven by purpose. Today, the development of advanced materials for health and well-being demands a truly multidisciplinary approach. In some intensive care units, engineers and clinicians already work side by side, translating laboratory discoveries into lifesaving technologies. This collaboration recalls an earlier vision of science as a unified endeavour, where boundaries between disciplines dissolve, and the pursuit of understanding becomes the shared gold standard.

## Key material properties and current performance in intensive care units

From biocompatible polymers that line vascular catheters to sensor-integrated surfaces that respond to physiological change, materials have become active participants in the care environment. Their design reflects a shared goal with intensive care itself — to sustain function under conditions of extreme fragility, and to extend the limits of what the human body can endure. Yet, as we embrace these innovations, it is worth reflecting on how the materials we create increasingly resemble the organs and functions they are meant to assist.

Sepsis continues to represent an important mortality and morbidity cause in intensive care units, multiple lines of evidence proving that early diagnosis and treatment can improve the outcome of such patients. [3]. The concept of organ dysfunction employed in the definition of sepsis refers to a complex alteration of biological processes occurring at cellular and metabolic levels [4]. In this context, advanced materials with antimicrobial and biosensing properties hold significant promise. Surface coatings capable of resisting bacterial adhesion, polymers that release antimicrobial agents in a controlled manner, and sensor-integrated materials that detect metabolic or inflammatory changes in real time collectively transform sepsis management. Such materials not only prevent infection but also serve as early diagnostic tools, aligning material science innovation with the critical care imperative of timely recognition and intervention. Advanced materials such as nanoparticles, coatings, and antimicrobial polymers are now being used to coat surfaces like bed rails, monitors etc. These materials not only prevent bacterial colonization but also reduce the need for constant disinfection, thus lowering cross-contamination risks. Additionally, biocompatible polymers like polylactic acid are used in devices that come into direct contact with patients, reducing the likelihood of immune rejection or inflammation, while improving patient outcomes and comfort.

Advanced materials also contribute to the development of wearable and embedded monitoring systems. Materials like flexible electronics and conductive fabrics are integrated into ICU garments or bedding to continuously monitor vital signs. These systems provide real-time data without the need for intrusive equipment, improving patient mobility and reducing discomfort. Novel, smart materials respond dynamically to physiological changes. For example, thermo-responsive polymers can change their properties based on body temperature, allowing for drug release or temperature regulation. This is particularly valuable in managing fever or hypothermia in critically ill patients.

The COVID-19 pandemic emphasized the need for reliable and efficient respiratory support systems. Advanced materials such as graphene-based membranes have been employed to improve the filtration and durability of ventilators and oxygen delivery systems. High-efficiency materials can effectively remove airborne pathogens, protecting both patients and healthcare workers. Moreover, the development of lightweight, durable materials for respiratory masks and helmets enhances comfort and long-term wearability, which is crucial for patients needing prolonged respiratory assistance.

Finally, many pieces of ICU equipment—such as patient beds, now incorporate advanced composite materials. Carbon fibre-reinforced plastics and lightweight metal alloys reduce the overall weight of equipment, improve manoeuvrability, and enhance structural integrity. This leads to faster emergency responses and easier repositioning of patients, which is essential in critical care scenarios.

Beyond their direct and measurable impact on ICU procedures, these material innovations carry subtler, yet equally meaningful, benefits for patients’ emotional well-being. Comfort, safety, and reliable monitoring before and after major interventions can ease the anxiety that so often accompanies critical illness. The anticipation of surgery, the procedure itself, and the long path of recovery are recognized sources of profound emotional strain, sometimes leading to post-traumatic stress or cardiovascular complications [5]. By creating environments and devices that feel safer and more responsive, advanced materials can help reduce this burden, thus supporting not only survival, but also the psychological resilience that underlies true recovery.

## Enabling the next generation of advanced materials through bioactivity

Some already existing outstanding features in advanced biomedical material are (i) the biocompatibility and biodegradability of some polymers, ceramics, and composites, designed to be safely absorbed by the body, reducing the need for repeated procedures, for example, for stents or drug delivery systems; (ii) biosensing of biomarker detection (e.g. graphene biosensors), which allows rapidly identifying substances such as lactic acid, crucial for diagnosing sepsis and monitoring the condition of critically ill patients; (iii) lightweight and non-invasiveness for wearable sensors (e.g. accelerometers) that continuously monitor patients’ activity and movement, providing rich data on their health progress; (iv) nanoscale structured materials such as nanoparticles, for drug carriers that protect the treatment in the bloodstream and release substances in a targeted manner, maximizing efficacy and minimizing side effects; (v) strength and lightness for mobility, frequent repositioning, and ergonomics, as well as to withstand constant cleaning, disinfection, and mechanical stress and (vi) multi-functional materials that present excellent mechanical flexibility, biocompatibility and stability, together with other features like transforming ambient stimuli into electric potentials by implementing medical sensing applications that maximally match present needs [6].

To enable future generation of ICU advanced materials through bioactivity we must assemble complex materials according to their functional priorities, by an application-oriented approach. Biointegration extends beyond biocompatibility, representing an active and continuous cooperation between an artificial material/system and the body. In the context of intensive care applications, biointegration is particularly critical, as it enables functional synergy between biomaterials and human tissues, whether dermal or internal [7].

In the development of advanced materials for ICU applications, the goal is to create multifunctional systems that enhance patient safety, prevent infections, and ensure biocompatibility across diverse medical devices. For example, nanoparticle coatings used in catheters to inhibit biofilm formation and infection can also be applied to wound dressings, where their antibacterial and biocompatible properties promote tissue healing. Selecting such cross-application materials involves identifying shared functional requirements—such as antimicrobial activity, hemocompatibility, or controlled release—and adapting fabrication technologies like plasma-assisted deposition or electrospinning to fine-tune surface chemistry, flexibility, and durability. This cross-functional strategy fosters the creation of ICU materials that seamlessly integrate infection prevention, biological compatibility, and mechanical performance across different clinical uses.

Despite the significant benefits, implementing new technologies in the ICU presents several challenges like:

- difficulty in combining good mechanical properties with biocompatibility and bioactivity; most of the resistant polymers (thermosets), for example, are not bioactive; on the opposite, thermoplastics although not robust, they are highly biocompatible, sustainable, and they enable biointegration
- high costs, since the initial investment in new materials and technologies, software, and IT infrastructure can be substantial, as for instance in the case of 3D printing technologies for rapid personalized solutions in ICUs
- ethical concerns surrounding the validation of novel materials through complex required protocols that are not yet accurately described.

Even so, ongoing interdisciplinary collaboration, continued research, and thoughtful implementation strategies promise to unlock the full potential of these innovations. For those of us designing these materials, the ICU represents more than a place of crisis — it is a testing ground for empathy in engineering. Every new polymer, coating, or sensor ultimately asks the same question: can we build technologies that understand fragility as deeply as they promise resilience?

## Ethical reflection

Ultimately, innovation in critical care must serve the patient—not the machine. As we embrace new technologies and materials, progress must be matched by the refinement of our testing and accreditation processes. Faster, more adaptive evaluation protocols and increasingly sophisticated experimental setups are essential to ensure that promising innovations reach the bedside safely and effectively. Above all, technological progress should enhance—not replace—the human presence at the heart of care.

## Conclusions and future outlooks

The synergy between engineering and medicine in the ICU environment continues to push the boundaries of what is possible in life-saving care. Looking ahead, the use of regenerative biomaterials and 3D-printed medical components is expected to revolutionize ICUs. Bioengineered tissues, such as artificial skin or vascular grafts, are being developed using biodegradable scaffolds and stem-cell-compatible materials. In critical cases involving trauma or burn injuries, these materials may soon be applied directly in the ICU setting for rapid intervention. 3D printing with advanced polymers and hydrogels also allows for the on-demand production of customized medical tools, prosthetics, or airway support structures. This adaptability is particularly useful in resource-limited or emergency scenarios. The future of ICU technology is focused on creating seamlessly integrated, human-centric systems. This requires collaboration among clinicians, data scientists, and industry leaders to ensure that technology is validated, equitable, and ultimately improves patient care without compromising the essential human connection in medicine.
